# Myocarditis Caused by Human Parechovirus in Adult

**DOI:** 10.3201/eid2309.161256

**Published:** 2017-09

**Authors:** Khai Lin Kong, Jillian S.Y. Lau, Su Mei Goh, Heather L. Wilson, Mike Catton, Tony M. Korman

**Affiliations:** Monash University, Clayton, Victoria, Australia (K.L. Kong, J.S.Y. Lau, S.M. Goh, T.M. Korman);; Monash Health, Clayton (K.L. Kong, J. Lau, S.M. Goh, T.M. Korman);; Peter Doherty Institute for Infection and Immunity, Melbourne, Victoria, Australia (H.L. Wilson, M. Catton)

**Keywords:** human parechovirus, viruses, myocarditis, Australia

## Abstract

The infectious etiology of myocarditis often remains unidentified. We report a case of myocarditis associated with human parechovirus (HPeV) infection in an adult. HPeV is an emerging pathogen that can cause serious illness, including myocarditis, in adults. Testing for HPeV should be considered in differential diagnosis of myocarditis.

Infections with human parechovirus (HPeV) are rarely reported in adults. We report a case of myocarditis associated with HPeV infection in an adult.

## The Study

During the summer of 2015, a 26-year-old man in Victoria, Australia, was admitted to Casey Hospital (Berwick, VIC, Australia) because of 4 days of fever, rigors, headache, dry cough, sore throat, myalgia, and a history of erythematous macular rash on arms bilaterally that had resolved by the time of admission. The patient smoked cigarettes and reported use of methamphetamines, but no other medical history was reported. He lived in a rural area but had no close contact with animals. He lived with 3 young children, including an 8-week-old infant who had recently had otitis externa.

At admission, he was febrile (temperature 38.2°C) and had sinus tachycardia (<130 beats/min). Results of a physical examination were otherwise unremarkable. Peripheral blood lymphocyte count was 0.70 × 10^9^ cells/L (reference range 1–4 × 10^9^ cells/L), C-reactive protein level 111 mg/L (reference value <5 mg/L), erythrocyte sedimentation rate 94 mm/h (reference value <10 mm/h), serum bilirubin level 49 μmol/L (reference value <20 μmol/L), and albumin level 24 g/L (reference range 35–45 g/L).

Microscopic analysis of cerebrospinal fluid (CSF) showed 2 × 10^6^ polymorphonuclear cells/L, 2 × 10^6^ lymphocytes/L, a total protein level of 0.5 g/L (reference range 0.1–0.3 g/L), and glucose and lactate levels within reference ranges. Blood and CSF cultures showed no bacterial growth. Because of a low leukocyte count, molecular studies for viruses (including enterovirus) were not performed for the CSF sample.

Fever and tachycardia persisted for 5 days and chest discomfort and dyspnea developed. A transthoracic echocardiogram showed a mildly dilated left ventricle with an ejection fraction of 15%. There were no valvular vegetations. Peak creatine kinase level was 713 U/L (reference value <230 U/L), and troponin level was 15.28 μg/L (reference value <0.080 μg/L).

The patient was given intravenous benzylpenicillin and oral doxycycline as empirical therapy for possible bacterial infection; Q fever and leptospirosis were considered possible diagnoses. Fever and chest discomfort improved, and he was discharged 7 days after admission. Two weeks later, the patient was well and had minimal dyspnea.

Throat swab specimens were obtained on day 6 of illness, and rectal swab specimens were obtained on day 8 of illness. Specimens were tested for enterovirus and HPeV RNA by reverse transcription PCR (RT-PCR) and primers specific for the highly conserved 5′ untranslated region (*1*) (details for HPeV primers and probes are available on request). HPeV was detected in the throat swab specimen, but not the rectal swab specimen.

We attempted molecular typing of HPeV by using the method of Papadakis et al. (*1*) and primers AN353, AN355, AN357, AN358, and AN369 described by Nix et al. (*2*). However, typing was not successful because of low copy numbers, probably caused by specimens being collected late in the illness.

Multiple investigations showed no other infectious causes of myocarditis. Serologic results were negative for previous or recent infections with hepatitis A, B, and C viruses and HIV, as well as *Leptospira* spp., *Coxiella burnetii*, rickettsia, *Treponema pallidum*, and *Toxoplasma* spp. Serologic analysis showed evidence of previous infections with cytomegalovirus and Epstein-Barr virus. However, a convalescent-phase serum sample was not available for additional serologic testing.

A multiplex PCR (Respiratory Pathogens B; AusDiagnostics, Beaconsfield, NSW, Australia) was performed for a nasopharyngeal swab specimen. Results were negative for influenza A virus; A(H1N1)pdm09 virus; influenza B virus; respiratory syncytial virus; rhinoviruses/enterovirus; human parainfluenza virus 1, 2, and 3; adenovirus (groups B, C, E, some A, D); human metapneumovirus; *Bordetella pertussis* and *B. parapertussis*; *Legionella pneumophila* and *L. longbeachae*; *Mycoplasma pneumoniae*; and *Chlamydia/Chlamydophila* spp. (including *C. psittaci*, *C. pneumoniae*, and *C. trachomatis*).

## Conclusions

HPeVs were previously classified as a subgenus of echoviruses (*3*). Echovirus subtypes 22 and 23 were renamed HPeV type 1 and 2; sixteen different types of HPeV thus far have been identified. Serosurveillance studies showed that by 2 years of age, <90% of children are infected with >1 type of HPeV (*3*). Infections with human parechoviruses show various clinical manifestations, notably sepsis-like disease and encephalitis in infants. A recent large outbreak of HPeV type 3 infections in infants was reported in Australia (*4*).

HPeV infections in adults are rarely reported. Mizuta et al. reported 22 adults with myalgia, muscular weakness, sore throat, orchiodynia, and increased levels of creatine phosphokinase; 14 had HPeV type 3 infections confirmed by virus isolation, positive RT-PCR results for throat swab or stool specimens, or serologic analysis (*5*). HPeV was also reported to be associated with flaccid paralysis and diarrheal illness in adults (*6**,**7*).

The rarity of HPeV infection in adults could be related to immunity conferred by previous exposure during childhood to HPeV. Few seroprevalence data are available for HPeV infections in adults. However, as part of an investigation of infant deaths associated with HPeV type 3 in Wisconsin, USA, limited serologic testing of 59 adults demonstrated that infections were not common, suggesting that either HPeV3 was a new pathogen being introduced to this community, or that there was waning immunity, which made antibody titers difficult to detect in adults (*8*).

The lack of documented reports of HPeV infection in adults could also be caused by lack of widespread testing for adults. HPeV RNA is not detected by routine enterovirus PCRs and requires additional HPeV testing. The Victorian Infectious Diseases Reference Laboratory (Melbourne, VIC, Australia) routinely tests specimens for enterovirus and HPeV when a request is made, regardless of the age of the patients. During January 2015–May 2016, this laboratory tested 3,525 specimens for HPeV, of which 1,425 (40%) were obtained from adults. HPeV was detected by RT-PCR in 5 (0.35%) of 1,425 specimens: 2 in throat swab specimens, 2 in blood, and 1 in CSF. In comparison, 286 (13.6%) of 2,100 specimens from persons <18 years of age were positive for HPeV; most (271, 94.8%) were from children <1 year of age. This finding suggests that, although increased testing for HPeV could increase the detection rate of HPeV infection in adults, it is an uncommon infection in the adult population. This finding is consistent with results of a study from a reference laboratory in Scotland that tested 3,739 CSF samples from persons of all ages and found that although enteroviruses were common in adults, HPeV infections were found exclusively in young infants (*9*).

Enteroviruses are recognized as a major cause of acute myocarditis and are associated with <14% of cases (*10*). Myocarditis associated with HPeV infections is rarely reported (Table). This disease has been reported in 3 children <2 years of age and 1 adolescent. Two of the patients were immunosuppressed, 1 of whom died. A study of 109 patients infected with echovirus 22 (now HPeV subtype 1) in Sweden included a case of myocarditis in a child; virus was isolated from a stool sample and a major increase in antibody titer was observed (*11*).

**Table:** Characteristics of 5 patients with myocarditis caused by infection with human parechovirus*

Patient (reference)	Age/sex	Underlying disease	Clinical features	Sample in which virus was detected	Subtype	Echocardiographic finding	Therapy	Outcome
1 (*11*)	NA/M	NA	NA	Stool, blood	1†	NA	NA	NA
2 (*13*)	14 mo/M	Congenital AGG	Myocarditis	Myocardium, pericardial fluid	1†	NA	None	Died
3 (*14*)	6 wk/M	None	Myocarditis	Stool	1†	NA	None	Survived
4 (*12*)	16 y/F	SLE, rituximab-induced HGG	Myocarditis, encephalitis	Myocardium, CSF, stool	3	Biventricular dysfunction, LVEF 13%	IVIG	Survived, prolonged neurologic recovery
5 (this study)	26 y/M	None	Myocarditis	Throat swab specimen	Unknown	Dilated left ventricle, LVEF 15%	None	Survived, Well at 6-mo follow up

*AGG, agammaglobulinemia; CSF, cerebrospinal fluid; HGG, hypogammaglobulinemia; IVIG, intravenous immunoglobulin; LVEF, left ventricular ejection fraction; NA, not available; SLE, systemic lupus erythematosus. †Previously known as echovirus subtype 22.

There is no proven effective therapy for HPeV infection. Intravenous immunoglobulin (IVIG) was used for 2 patients (Table). IVIG has been used for treatment of enterovirus infections, particularly in immunocompromised patients (*15*), but the efficacy of IVIG might be limited for treatment of HPeV infection because of low seroprevalence in adults (*8*).

In summary, we report a case of myocarditis associated with HPeV infection in an adult. A large proportion of cases of myocarditis has no identified infectious cause. Thus, testing of throat swab, stool, and blood specimens for HPeV should be considered for adults with myocarditis. HPeV is an emerging pathogen that can cause major illness, including myocarditis, in adults.
